# Arrhythmic Risk Stratification among Patients with Hypertrophic Cardiomyopathy

**DOI:** 10.3390/jcm12103397

**Published:** 2023-05-10

**Authors:** Francesco Santoro, Federica Mango, Adriana Mallardi, Damiano D’Alessandro, Grazia Casavecchia, Matteo Gravina, Michele Correale, Natale Daniele Brunetti

**Affiliations:** 1 Cardiology Unit, Department of Medical and Surgical Sciences, University of Foggia, 71122 Foggia, Italy; 2 Radiology Unit, University Polyclinic Hospital of Foggia, 71100 Foggia, Italy

**Keywords:** arrhythmias, genetic testing, hypertrophic cardiomyopathy, HCM, risk score, sudden cardiac death

## Abstract

Hypertrophic cardiomyopathy (HCM) is a cardiac muscle disorder characterized by generally asymmetric abnormal hypertrophy of the left ventricle without abnormal loading conditions (such as hypertension or valvular heart disease) accounting for the left ventricular wall thickness or mass. The incidence of sudden cardiac death (SCD) in HCM patients is about 1% yearly in adults, but it is far higher in adolescence. HCM is the most frequent cause of death in athletes in the Unites States of America. HCM is an autosomal-dominant genetic cardiomyopathy, and mutations in the genes encoding sarcomeric proteins are identified in 30–60% of cases. The presence of this genetic mutation carries more than 2-fold increased risk for all outcomes, including ventricular arrhythmias. Genetic and myocardial substrate, including fibrosis and intraventricular dispersion of conduction, ventricular hypertrophy and microvascular ischemia, increased myofilament calcium sensitivity and abnormal calcium handling, all play a role as arrhythmogenic determinants. Cardiac imaging studies provide important information for risk stratification. Transthoracic echocardiography can be helpful to evaluate left ventricular (LV) wall thickness, LV outflow-tract gradient and left atrial size. Additionally, cardiac magnetic resonance can evaluate the prevalence of late gadolinium enhancement, which when higher than 15% of LV mass is a prognostic maker of SCD. Age, family history of SCD, syncope and non-sustained ventricular tachycardia at Holter ECG have also been validated as independent prognostic markers of SCD. Arrhythmic risk stratification in HCM requires careful evaluation of several clinical aspects. Symptoms combined with electrocardiogram, cardiac imaging tools and genetic counselling are the modern cornerstone for proper risk stratification.

## 1. Introduction

Hypertrophic cardiomyopathy (HCM) is a cardiac muscle disorder characterized by generally asymmetric abnormal hypertrophy of the left ventricle without abnormal loading conditions (such as hypertension or valvular heart disease) accounting for the left ventricular wall thickness or mass [1,2]. In most of the cases, mutations in the genes encoding sarcomeric proteins are an autosomal dominant trait, responsible for the observed abnormality [1,2]. It is a quite common disease with an estimated prevalence of 1:500 in the general population, whereas in children the prevalence is much lower [3,4,5].

Sudden cardiac death (SCD), mainly caused by potentially fatal and unpredictable malignant ventricular arrhythmias (VAs) is the most adverse complication of HCM [6,7], with an annual incidence of SCD of approximately 1% in adult HCM patients and far higher in subgroups, such as pediatric HCM patients [8]. It may occur as the initial disease presentation, frequently in asymptomatic or mildly symptomatic young people and even athletes [9].

Several mechanisms predispose HCM patients to re-entrant VA. Genetic and myocardial substrate, including fibrosis and intraventricular dispersion of conduction, disruption of intercalated discs and myofibrillar disarray, ventricular hypertrophy and microvascular ischemia, increased myofilament calcium sensitivity and abnormal calcium handling all play a role as arrhythmogenic determinants [10,11,12]. Precipitating factors include intense physical exertion and participation in competitive sport or intrinsic features of the disease, such as left ventricular outflow obstruction, which can trigger life-threatening ventricular tachyarrhythmias [7].

Pharmacologic therapy has not proved to be effective alone in providing protection from SCD if compared to implantable cardioverter defibrillators (ICDs). If ICD implantation in secondary prevention is a well-established practice, the real challenge stands in identifying a special subset of HCM patients who are at high risk of SCD prior to a first event and would benefit from an ICD [13]. Therefore, systematic arrhythmic risk stratification at initial evaluation and then periodically is strongly recommended by current clinical guidelines [14]. The aim of this review is to highlight and discuss the most important factors associated with SCD in HCM to guide more comprehensive and exhaustive arrhythmic risk stratification.

## 2. Demographic and Clinical Characteristics

SCD can occur independently both in male and female HCM patients. However, the role of gender is still a matter of debate. While male patients more frequently show fibrosis on histological examination and consequently usually suffer more often from VA, no study has succeeded in proving a significant association between sex and SCD [2]. Age is a crucial point in SCD related to HCM. Given the potential lifetime risk of SCD in HCM patients, the incidence of SCD is far higher in adolescence and early adulthood, especially in those under the age of 35, with HCM being the most frequent cause of death in athletes in the US [15]. It is generally infrequent in patients older than 60 years as proved by Spirito et al., who demonstrated a significant reduction in SCD risk with increasing age [16].

Family history of SCD (FHSCD) is one of the major factors associated with arrhythmic risk in HCM patients. Personal anamnesis positive for FHSCD events, especially if multiple or occurring at a younger age, carries an increased risk of SCD among individuals of approximately 20% if compared to family without an obvious family history [17]. Nonetheless, definitions of FHSCD may vary considerably—with FHSCD generally considered when one or more first-degree relatives under 40 or 50 years of age dies incidentally within 1 h (witnessed) or 24 h (asymptomatic observation) after the symptom appears—thus influencing the effective individual’s risk [18]. However, the average hazard ratio of FHSCD (irrespective of definition) was 1.27 (95% confidence interval (CI) 1.16–1.38) in a systematic review [19]. Different mechanisms responsible for FHSCD have been pointed out, although it is often a dilemma to identify the exact cause of death in the relatives. Considering the genetic nature of the disease, with affected relatives sharing the same genetic defect and with specific mutations associated with a worse prognosis, the extent of the environmental exposure cannot be adequately measured, all translating into a significant variability as to the genotype-phenotype correlation [20,21].

Syncope is defined as a temporary loss of consciousness secondary to transient global cerebral hypoperfusion. Spirito et al. defined unexplained syncope as syncope “of unknown origin, when it occurred in circumstances not clearly consistent with a neurally mediated event, i.e., without apparent explanation at rest or during ordinary daily activities, or during an intense effort” [16]. Multiple studies have shown that there is a significant association between unexplained syncope and SCD. In addition, since there are several causes of syncope in HCM including arrhythmias (sustained ventricular arrhythmias, supraventricular tachycardias, atrial fibrillation, brady-arrhythmias), exercise-related left ventricular outflow-tract obstruction (LVOTO), mitral regurgitation, ischemia and microvascular angina, although even neurally mediated syncope (vasovagal and situational) and orthostatic hypotension are possible, it is very important to deeply analyze the clinical context in which the syncope takes place [22,23]. Given such a high possibility of causes, clues from the patient and the witnesses may help. Additional attention should also be paid to exertional or recurrent syncope if it occurs in the young and in the recent past (<6 months) [24].

There is no particular association between NYHA functional class and the risk of SCD, with SCD being reported in all NYHA classes [25]. However, several factors are involved in functional limitations in HCM including the degree of diastolic dysfunction, LVOTO, cardiac ischemia and microvascular angina and atrial arrhythmias, especially atrial fibrillation (Table 1) [26].

## 3. Non-Invasive Markers (ECG, Systolic Blood Pressure (SBP) Response to Exercise, Cardiopulmonary Exercise Test)

### 3.1. Electrocardiogram and Holter ECG

The twelve-lead electrocardiogram (ECG) has been used to evaluate electrophysiological abnormalities in HCM, and some of the ECG parameters including microvolt T-wave alternans, T-peak/T-end interval, fragmented QRS complexes and QT duration were found to be well correlated with myocardial fibrosis and arrhythmic events [27,28]. Given its fast and easy-to-perform evaluation, surface ECG analysis should always be included in each patient evaluation.

The microvolt T-wave alternans (MTWA) consists of microscopic alternans measured in microvolts on every heartbeat and is evidenced in the amplitude or the morphology of the T-wave. The alternation of T-waves in patients with HCM may possibly be explained as a result of inhomogeneous action, potential propagation and heterogeneous repolarization due to abnormal cell-to-cell conduction [29]. Özyılmaz S et al. tried to assess the relationship between the presence of MTWA and the predicted 5-year risk of SCD among patients with hypertrophic cardiomyopathy (HCM). Authors found that patients with MTWA on a Holter ECG had higher risk of ventricular arrhythmias [30].

Akboğa et al. [31] tried to evaluate the electrocardiographic T-wave peak to end interval (Tp–e) and Tp–e/QT corrected (QTc) ratio among patients with HCM. The patients were divided into two groups: those with VA (*n* = 26) and those without VA (*n* = 40). Tp–e interval was significantly longer and Tp–e/QTc ratio were significantly higher in HCM patients with VA.

The fragmented QRS (fQRS) complex reflects intraventricular conduction delay and may then be a superficial marker for myocardial fibrosis. Myocardial fibrosis in HCM patients usually has a patchy distribution, and it may not always be detected by pathological Q-waves on a 12-lead ECG. Konno T. et al. demonstrated that fQRS may have a substantially higher sensitivity and diagnostic accuracy if compared to pathological QRS in detecting myocardial fibrosis in HCM patients [32]. Considering its strong association with myocardial fibrosis, according to Ki-Woon Kang et al., fQRS may be a good candidate marker for prediction of VA in HCM patients [33].

Non-sustained ventricular tachycardia (NSVT), defined as three or more consecutive ventricular beats with a frequency of at least 120 beats per minute (bpm) lasting for less than 30 s, and not causing hemodynamic instability, is a very common finding in HCM patients and is often documented in Holter monitoring [34].

NSVT is more frequent with increasing hypertrophy, which may automatically reflect an increased grade of fibrosis and myofibrillar disarray, which themselves are useful predictors of the intrinsic arrhythmic risk of the disease [35,36].

Maron et al. [37] and McKenna et al. [38] showed that in HCM patients NSVT is more common in SCD patients. However, several studies examined the relationship between NSVT and SCD in HCM patients with a prevalence of NSVT ranging between 17% and 32% due to a non-uniform NSVT definition [39,40]. Even though a high incidence rate of NSVT (approximately 20–30%) in HCM patients over the age of 40 is reported, the risk of SCD linked to NSVT seems to be lower in older patients. According to Monserrat et al., a 4-fold increase in the risk of SCD was observed in patients aged ≤30 years with NSVT, (univariable HR, 4.35; 95% CI, 1.54–12.28; *p* = 0.006), but no effects were observed in older patients (univariable HR, 2.16; 95% CI, 0.82–5.69; *p* = 0.1), with frequency, duration and rate of NSVT not having predictive value [41].

### 3.2. Systolic Blood Pressure (SBP) Response to Exercise

An abnormal blood pressure response to exercise testing, defined as either a drop of at least 20 mmHg during effort or a failure to increase from rest to peak exercise by at least 20 mmHg, is a quite common finding in HCM patients, occurring in more than one out of three HCM patients [42].

Several mechanisms have been studied and are believed to be responsible for this phenomenon, including a hemodynamic hypothesis with an inappropriate drop in systemic vascular resistance, despite an appropriate increase in cardiac output, and/or LVOTO [43,44]. Although the prognostic role of systemic blood pressure response to exercise is still debated [45], it has been introduced as an additional risk factor from the European society of Cardiology (ESC) SCD 2022 guidelines and should be evaluated among patients with an intermediate SCD risk.

### 3.3. Cardiopulmonary Exercise Test

Cardiopulmonary exercise testing (CPET) data have been shown to improve the risk stratification of patients with heart failure. In the context of hypertrophic cardiomyopathy, a reduced oxygen consumption peak, an increased ventilation/carbon dioxide production slope and chronotropic incompetence correlate with a worse prognosis [46].

Two research groups of Magri et al. [47] and Masri et al. [48] showed that a reduced VO2 peak (i.e., <50%) and high VE/VCO2 slope are associated with overall mortality and SCD in HCM. Recently, the 2020 Guidelines on sports cardiology from ESC included in the indications for the execution of CPET the evaluation of exercise-induced symptoms or arrhythmias and the assessment of systolic blood pressure changes during exercise [49] (Table 2).

## 4. Role of Genetics

HCM is an autosomal-dominant genetic cardiomyopathy, and mutations in the genes encoding sarcomeric proteins are identified in 30–60% of index cases. Eight sarcomeric gene mutations are the most common described in literature for HCM: *MYBPC3*, *MYH7b*, *MYL2*, *MYL3*, *TNNT2*, *TNNI3*, *TPM1* and *ACTC1* (Table 3) [14]. The rate of major adverse cardiovascular events (MACEs) and premature death is significantly higher in patients carrying mutations in the genes encoding sarcomeric proteins than in negative ones [50]. Several analyses have shown that the presence of a mutation in the gene encoding sarcomeric proteins carries a more than 2-fold increased risk for all outcomes, including ventricular arrhythmias, which are more frequent in this group of patients [51]. Mutation of the MYH7 gene is associated with a more aggressive phenotype, characterized by younger onset age, higher degree of left ventricular hypertrophy and higher risk of SCD, resulting in a poor prognosis [52]. This group of patients also suffers from a higher incidence of atrial fibrillation (AF), which is associated with other risk factors such as left atrium (LA) enlargement, left ventricle (LV) wall thickness and LV outflow-tract obstruction. Compared with MYH7 gene mutation, patients with MYBPC3 mutation usually develop the disease at a later age and have a favorable progression of the disease although they are associated with a non-negligible risk of SCD compared to the healthy population. Because of the high risk, intense exercise should be routinely discouraged, especially in patients with the MYH7 gene mutation. Mutations in the TNNT2 gene may manifest with mild LV wall thickening but have more severe myocyte disarray, younger patients and a high incidence of SCD [53]. Given the clinical profile, patients carrying mutations in the genes encoding sarcomeric proteins should benefit from more intensive clinical surveillance. The ESC 2022 guidelines recommend genetic counselling and testing in all HCM patients (Class I, Level B), emphasizing the value of the genotype in guiding clinical management and determining prognosis [14]. They also represent an additional risk factor for HCM patients at intermediate SCD risk [14].

## 5. Cardiac Imaging (Echocardiogram and Cardiac Magnetic Resonance)

**Figure 1:**
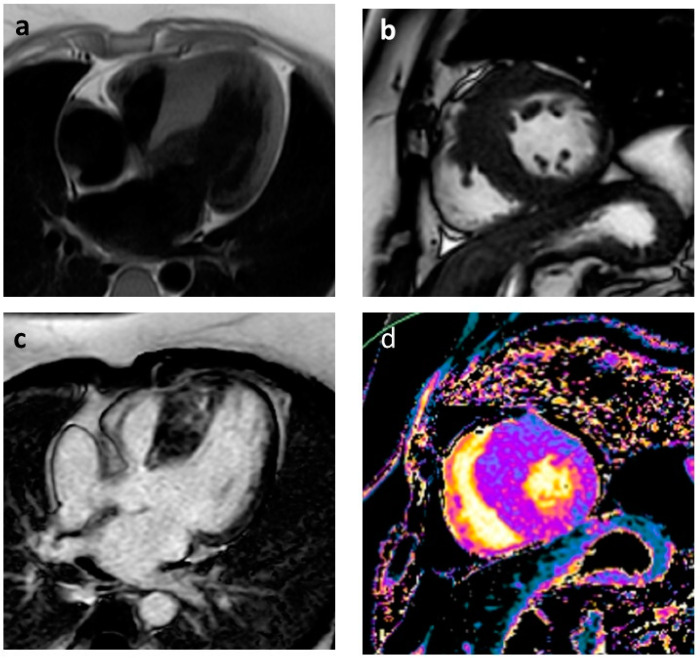
Asymmetric hypertrophic cardiomyopathy; thickening of the interventricular septum which can be evaluated with the T1-TSE four-chamber sequences (**a**); no evident edema in the short axis T2-STIR sequence (**b**); irregular deposits of mesocardial paramagnetic contrast medium in PSIR-TFE sequences in four chambers for the study of “late gadolinium enhancement” (**c**); T1 mapping analysis showing a diffuse increase in signal of the various segments of the walls of the left ventricle as from minimal diffuse interstitial fibrotic deposits (**d**).

**Figure 2:**
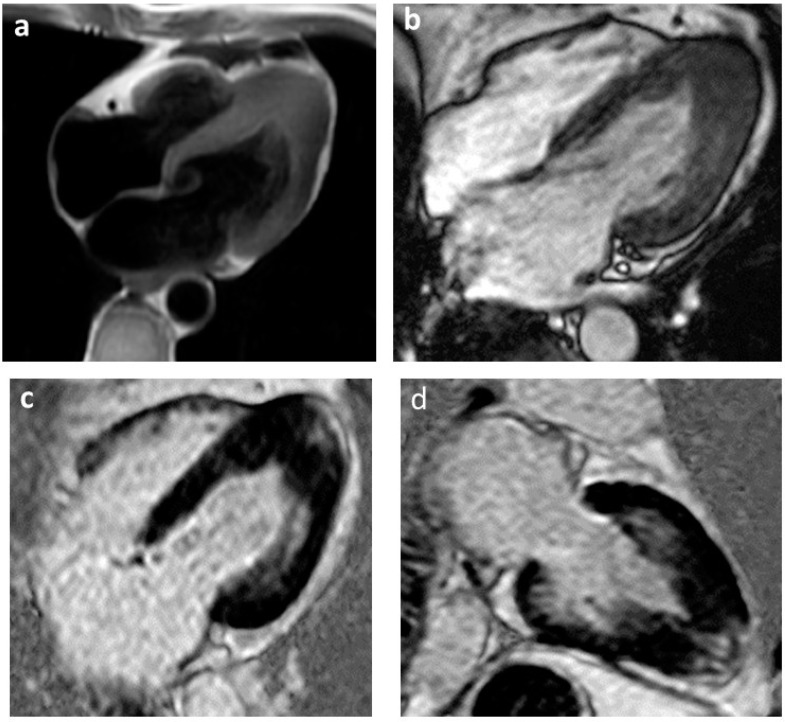
Apical hypertrophic cardiomyopathy with concentric thickening of the left ventricular wall at four-chamber T1-TSE sequences (**a**); absence of edema at four-chamber view T2-STIR sequence (**b**); slight increase in meso-subendocardial signal evident in PSIR-TFE sequences in four and two chambers for the study of “late gadolinium enhancement” (**c**,**d**) compatible with minimal fibrotic deposits.

Cardiac imaging plays a crucial role in management of HCM patients. The gold standard for diagnosis of HCM, assessment of treatment efficacy and prognosis is transthoracic echocardiography supported by cardiac magnetic resonance (CMR) imaging, which plays a central role in the diagnostic process. In 2014, the ESC validated an SCD risk prediction model that provides a 5-year SCD risk score for HCM patients. Echocardiography provides three of the seven parameters required in the 5-year SCD risk stratification score (LV wall thickness, LA size, LVOT gradient) [54]. LV hypertrophy is associated with increasing prevalence of NSVT and exercise-induced VAs. Several studies showed a significant correlation between severe hypertrophy of LV and SCD. Severe left ventricular hypertrophy (LVH) may contribute to SCD due to its effects on myocardial architecture, intramural small vessel disease and mass-to-coronary flow mismatch. A cut-off of maximum wall thickness ≥30 mm was used to indicate severe hypertrophy and was seen to be independently associated with SCD [2]. The LV wall thickness must be measured accurately at end-diastole, and elements attached to but not incorporated in the septum, such as papillary muscles, false tendons and right ventricular (RV) trabeculation, should be excluded because wall thickness could be overestimated. Left atrial diameter, quantified with echocardiography in the parasternal long axis, has been associated with SCD in HCM. AF and left atrial size may reflect the risk of SCD, as they may both relate to atrial remodeling secondary to increasing ventricular fibrosis, which makes the myocardium more susceptible to arrhythmias [7]. Diastolic dysfunction is common in HCM and results in elevated filling pressures and left atrial dilatation, so it is also a predictor of arrhythmic events. Patients with a restrictive diastolic filling pattern have adverse outcomes and should be observed closely. LVOTO is caused by systolic anterior movement of the mitral valve into the outflow tract, which creates a physical barrier impeding the flow of blood from the ventricle to the aorta during systole. Significant dynamic obstruction is defined as the presence of an instantaneous peak basal gradient ≥30 mmHg or after provocative maneuvers (Valsalva, standing and exercise) ≥50 mmHg [55]. Several studies reported a significant association between SCD and LVOTO. LVOTO can cause SCD either through a severe reduction in cardiac output or by myocardial ischemia due to the increase in left ventricular filling pressure, thus creating a substrate for ventricular arrhythmias. Although not included in the ESC risk calculator, additional factors, including LV systolic dysfunction, apical aneurysm, extensive LGE on CMR and presence of sarcomeric mutations, should be considered as possible modifiers of SCD risk [14]. Approximately 2–5% of patients with HCM, typically those with mid-ventricular hypertrophy, develop a left ventricular apical aneurysm associated with regional scarring. A higher incidence of clinical events during follow-up have been reported in this subgroup, including SCD and ventricular arrhythmia [6]. CMR allows accurate measurement of LV wall thickness, LV mass and LV ejection fraction and is the gold standard method for tissue characterization and volumetric evaluation of cardiac chambers. The extent of myocardial scarring on CMR has been shown to predict HCM-related adverse events. Myocardial fibrosis plays a central role in the genesis of arrhythmias through mechanisms of dispersion of electrical activity and formation of re-entry circuits that are responsible for the genesis of ventricular arrhythmias; this, as assessed by CMR, is independently associated with the occurrence of NSVT [56]. LGE is present in 65% of HCM patients, typically in a patchy mid-wall pattern in areas of hypertrophy and at the anterior and posterior RV insertion points (Figure 1 and Figure 2). A multicenter study found a linear correlation between the risk of SCD and amount of LGE. Extensive LGE on CMR defined as ≥15% of LV mass has been suggested as good predictor of SCD and appropriate ICD therapies in adults [57].

## 6. Programmed Electrical Stimulation for Risk Stratification

The role of programmed electrical stimulation (PES) to stratify arrhythmic risk in HCM patients is still controversial. Moreover, most of the studies on the topic date back to the 1980s and are difficult to apply nowadays as most of the studied patients undergoing PES would now be considered high risk by current guidelines and thus already eligible for ICD implantation [58].

The largest study, performed in the late 1980s, proved that induction of ventricular arrhythmias with aggressive PES resulted in a 5-year survival decrease [59]. Aggressive stimulation protocol was able to induce polymorphic ventricular tachycardia (VT) in 76% of inducible patients, with polymorphic VT being the most commonly induced arrhythmia. Geibel et al. studied the effect of PES in HCM patients with or without documented VA through a standardized stimulation protocol [60]. In HCM patients, a stimulation protocol with up to two extra stimuli was sufficient to identify patients with documented sustained monomorphic VT, although there may be some problems with specificity. The sample was very small, which prevented further conclusions from being drawn.

On the other hand, more recently, according to Gatzoulis et al., inducibility at PES predicts SCD or appropriate device therapy in HCM and non-inducibility is associated with prolonged event-free survival [61].

At the moment, no explicit consensus on when to perform PES in HCM patients for arrhythmic risk stratification has been approved, and PES is not considered for arrhythmic risk stratification in current guidelines due to its invasiveness and also due to the fact that VAs induced by PES are still considered non-specific [14].

## 7. Clinical Score

Despite the fact that SCD in HCM patients is a rare event, it still remains the most adverse and fearsome complication, especially considering that it often occurs in asymptomatic patients and without premonitory symptoms. As a result, identifying a special subset of HCM patients at increased risk for SCD in primary prevention is to be considered a great clinical challenge, and several studies over decades have tried to recognize major clinical risk markers to stratify HCM patients at high risk for SCD who would benefit from an implantable cardioverter defibrillator (ICD) [62]. In addition, both the risk stratification strategy and the spread of ICDs into clinical practice have contributed to cutting disease-related mortality rates. Therefore, the need for arrhythmic risk stratification became a prevalent issue and led the scientific community to develop clinical risk scores. Over the past 20 years, two major risk stratification systems have been incorporated in the clinical practice according to the American College of Cardiology/American Heart Association (ACC/AHA) [63] and the ESC [54] (Table 4).

ACC/AHA guidelines focus on a comprehensive analysis of non-invasive risk markers to identify patients most likely to benefit from an ICD in primary prevention, which is recommended to be performed at initial evaluation and every 1 to 2 years thereafter [63].

The American guidelines identify major risk factors for SCD: sudden death judged definitively or likely attributable to HCM in ≥1 first-degree or close relatives who are ≤50 years of age; massive LVH ≥ 30 mm in any LV segment; ≥1 recent episodes of syncope suspected by clinical history to be arrhythmic (i.e., unlikely to be of neurocardiogenic (vasovagal) etiology or related to LVOTO); LV apical aneurysm, independent of size; LV systolic dysfunction (EF <50%). According to these guidelines, if any of these major risk factors is present, ICD implantation is reasonable (class 2a indication).

If the decision to proceed to ICD implantation is still uncertain or the HCM patient does not seem to have increased risk of SCD after assessment of the previous risk factors, ICD may be considered in patients with extensive LGE determined by contrast-enhanced CMR imaging or NSVT present on ambulatory monitoring (class 2b indication in HCM patients aged ≥ 16 years; class 2a indication in HCM patients aged <16 years).

Moreover, additional parameters, including left atrial diameter and maximal LVOT gradient, may be considered to calculate an estimated 5-year SCD risk through a predictive risk score calculator available online (https://professional.heart.org/en/guidelines-and-statements/hcm-risk-calculator accessed on 15 February 2023) to assist shared decision-making between the physician and the patient for HCM patients ≥16 years old.

ESC guidelines [54] propose a more quantitative approach to SCD prediction through a score that predicts the 5-year risk for SCD. Seven factors have been included: age, LV wall thickness, LA size, LVOT gradient, NSVT, unexplained syncope and family history of SCD. Using a multivariable regression model, an online calculator has been created, and HCM patients are therefore stratified into a low (<4%), intermediate (with a risk of 4 to less than 6%) and high (≥6%) 5-year risk of SCD.

According to ESC Guidelines, in patients with a low 5-year risk of SCD, an ICD is generally not indicated, whereas in patients with a high 5-year risk, an ICD should be considered. In patients at intermediate risk, an ICD may be considered, taking into account the risks and benefits of ICD implantation as well as the patient preferences in a view of a more individualized approach.

The ESC risk score was later validated with variable results by several research groups [64,65].

Neither the AHA-HCM-SCD calculator nor the ESC-HCM Risk-SCD score have been validated in the following cohorts of patients and therefore should not be used in pediatric patients (<16 years), elite/competitive athletes, HCM associated with metabolic diseases (e.g., Anderson–Fabry disease) and syndromes (e.g., Noonan syndrome).

Regarding risk stratification for SCD in pediatric patients, important news came from the ESC guidelines of 2022 [14]. These guidelines introduce The HCM Risk-Kids score [66] that has been developed and externally validated [67] for children with HCM (1–16 years of age). It includes unexplained syncope, maximal LV wall thickness, large left atrial diameter, low LVOT gradient and NSVT (https://hcmriskkids.org accessed on 15 February 2023). In contrast to adults’ risk score, the age and family history of SCD did not improve its performance.

The American guidelines, however, do not yet accept a pediatric risk score. For the AHA/ACC 2020 guidelines on HCM, the decisions of ICD placement in pediatric patients must be based on individual judgment for each patient, taking into account all age-appropriate risk markers.

## 8. Future Perspectives

The increasing knowledge in several fields will provide additional insight for better risk stratification. Indeed, several novel approaches for left ventricular outflow tract may reduce arrhythmic risk. Apart from interventional therapy with surgery or radiofrequency [68], novel pharmacological treatment with selective and reversible inhibitors of the cardiac myosin ATPase have been demonstrated to provide improvement in exercise capacity, NYHA functional class and reduction in LVOT gradient [69]. Moreover, apart from cardiac magnetic resonance quantification of LV scars, novel software that evaluate scar features including border zone and conducting channels mass can better predict ICD intervention and therefore could be included in arrhythmic risk stratification [70]. All these data will be combined through artificial intelligence that could stratify different risk phenotypes [71].

## 9. Conclusions

Arrhythmic risk stratification in HCM requires careful evaluation of several clinical aspects. Symptoms combined with electrocardiogram, cardiac imaging tools and genetic counselling are the modern cornerstone for proper risk stratification.

## Figures and Tables

**Table 1:** Demographic and clinical characteristics associated with the risk of sudden cardiac death. Legend: CMR = cardiac magnetic resonance, FHSCD = family history of sudden cardiac death, LVOTO = left ventricular outflow-tract obstruction, NHYA = New York Heart Association, VA = ventricular arrhythmias.

Sex	no significant association	🟡 male patients show more fibrosis on CMR and experience VA more often
Age	strong association	🔴 especially in adolescence and early adulthood
FHSCD	strong association	🔴 particularly if multiple or occurring at younger ages
Unexplained syncope	strong association	🔴 additional attention required if exertional or recurrent
NYHA class	no particular association	🟡 consider functional limitations, including degree of diastolic dysfunction, LVOTO, microvascular angina and atrial fibrillation

**Table 2:** Non-invasive test and prognostic role in patients with hypertrophic cardiomyopathy.

Type of Test	Evaluation	Prognostic Role
Holter ECG	Non-sustained ventricular tachycardia	🔴 Can stratify arrhythmic risk
Exercise Testing	Systolic blood pressure during exercise test	🟡 Prognostic role is still debated
Cardiopulmonary Exercise Test	Vo2 peak$Ve/Vco2	🔴 Reduced Vo2 peak (<50%); High Ve/Vco2 slope is associated with overall mortality

**Table 3:** Sarcomeric gene mutation described in hypertrophic cardiomyopathy.

Gene	Population Frequency	Protein
		**Thick Myofilament Protein**
*MYBPC3*	25%	Myosin binding protein-
*MYH7* *B*	20%	Myosin heavy chain
*MYL2*	<1%	Regulatory myosin light chain
*MYL3*	<1%	Essential myosin light chain
		**Thin Myofilament protein:**
*TNNT2*	1.3%	Cardiac troponin T
*TNNI3*	1.3%	Cardiac troponin I
*TPM1*	>5%	Tropomyosin
*ACTC1*	<1%	Cardiac α-actin

**Table 4:** Clinical score proposed by European society of cardiology (HCM RISK-SCD) and American heart association (AHA-HCM-SCD) for sudden cardiac death risk stratification. Legend: CMR = cardiac magnetic resonance, LV = left ventricle, LA = left atrium, LVOT = left ventricular outflow tract, NSVT = non-sustained ventricular tachycardia, LGE = late gadolinium enhancement, SCD = sudden cardiac death.

Clinical Score for SCD in HCM	HCM RISK-SCD (2014)	AHA-HCM-SCD (2020)
Age	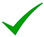	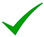
Family History Of SCD	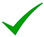	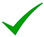
Syncope	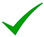	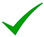
LV Apical Aneurysm		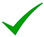
LV Systolic Dysfunction		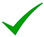
Maximal LV Wall Thickness	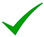	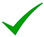
LA Size	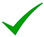	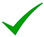
LVOT Gradient	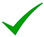	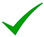
NSVT at Holter ECG	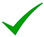	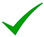
LGE at CMR		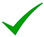

## Data Availability

Not applicable.

## Abbreviations

AF = atrial fibrillation; ACC/AHA = American College of Cardiology/American Heart Association; CMR = cardiac magnetic resonance; CPET = cardiopulmonary exercise testing; ESC = European Society of Cardiology; FHSCD = family history of SCD; fQRS = fragmented QRS; ECG = electrocardiogram; HCM = hypertrophic cardiomyopathy; ICD = implantable cardioverter defibrillator; LA = left atrium, LGE = late gadolinium enhancement; LV = left ventricle; LVH = left ventricular hypertrophy; LVOTO = left ventricular outflow-tract obstruction; MACE = major adverse cardiovascular events; MTWA= microvolt T-wave alternans; PES = programmed electrical stimulation; NSVT = non-sustained ventricular tachycardia; NYHA = New York Heart Association; SCD = sudden cardiac death; Tp–e = T-wave peak to end interval; VA = ventricular arrhythmia; VT = ventricular tachycardia

## References

[1] Elliott P., Andersson B., Arbustini E., Bilinska Z., Cecchi F., Charron P., Dubourg O., Kuhl U., Maisch B., McKenna W.J. (2008). Classification of the cardiomyopathies: A position statement from the european society of cardiology working group on myocardial and pericardial diseases. Eur. Heart J..

[2] O’Mahony C., Elliott P., McKenna W. (2013). Sudden Cardiac Death in Hypertrophic Cardiomyopathy. Circ. Arrhythm. Electrophysiol..

[3] Fananapazir L., Epstein N.D. (1995). Prevalence of Hypertrophic Cardiomyopathy and Limitations of Screening Methods. Circulation.

[4] Zou Y., Song L., Wang Z., Ma A., Liu T., Gu H., Lu S., Wu P., Zhang Y., Shen L. (2004). Prevalence of idiopathic hypertrophic cardiomyopathy in China: A population-based echocardiographic analysis of 8080 adults. Am. J. Med..

[5] Semsarian C., Ingles J., Maron M.S., Maron B.J. (2015). New Perspectives on the Prevalence of Hypertrophic Cardiomyopathy. J. Am. Coll. Cardiol..

[6] Yinga H., Su W.W., Xiaoping L. (2022). Risk factors of sudden cardiac death in hypertrophic cardiomyopathy. Curr. Opin. Cardiol..

[7] Jordà P., García-Álvarez A. (2018). Hypertrophic cardiomyopathy: Sudden cardiac death risk stratification in adults. Glob. Cardiol. Sci. Pract..

[8] Elliott P.M., Poloniecki J., Dickie S., Sharma S., Monserrat L., Varnava A., Mahon N.G., McKenna W.J. (2000). Sudden death in hypertrophic cardiomyopathy: Identification of high risk patients. J. Am. Coll. Cardiol..

[9] Maron B.J., Doerer J.J., Haas T.S., Tierney D.M., Mueller F.O. (2009). Sudden deaths in young competitive athletes: Analysis of 1866 deaths in the United States, 1980–2006. Circulation.

[10] Baudenbacher F., Schober T., Pinto J.R., Sidorov V.Y., Hilliard F., Solaro R.J., Potter J.D., Knollmann B.C. (2008). Myofilament Ca^2+^ sensitization causes susceptibility to cardiac arrhythmia in mice. J. Clin. Investig..

[11] Sepp R., Severs N.J., Gourdie R.G. (1996). Altered patterns of cardiac intercellular junction distribution in hypertrophic cardiomyopathy. Heart.

[12] Bahrudin U., Morikawa K., Takeuchi A., Kurata Y., Miake J., Mizuta E., Adachi K., Higaki K., Yamamoto Y., Shirayoshi Y. (2011). Impairment of ubiquitin-proteasome system by E334K cMyBPC modifies channel proteins, leading to electrophysiological dysfunction. J. Mol. Biol..

[13] Maron B.J., Haas T.S., Shannon K.M., Almquist A.K., Hodges J.S. (2009). Long-term survival after cardiac arrest in hypertrophic cardiomyopathy. Heart Rhythm.

[14] Zeppenfeld K., Tfelt-Hansen J., de Riva M., Winkel B.G., Behr E.R., Blom N.A., Charron P., Corrado D., Dagres N., de Chillou C. (2022). 2022 ESC Guidelines for the management of patients with ventricular arrhythmias and the prevention of sudden cardiac death. Eur. Heart J..

[15] Gersh B.J., Maron B.J., Bonow R.O., Dearani J.A., Fifer M.A., Link M.S., Naidu S.S., Nishimura R.A., Ommen S.R., Rakowski H. (2011). 2011 ACCF/AHA guideline for the diagnosis and treatment of hypertrophic cardiomyopathy: Executive summary: A report of the American College of Cardiology Foundation/American Heart Association Task Force on Practice Guidelines. Circulation.

[16] Spirito P., Autore C., Rapezzi C., Bernabò P., Badagliacca R., Maron M.S., Bongioanni S., Coccolo F., Estes N.M., Barillà C.S. (2009). Syncope and Risk of Sudden Death in Hypertrophic Cardiomyopathy. Circulation.

[17] McKenna W., Deanfield J., Faruqui A., England D., Oakley C., Goodwin J. (1981). Prognosis in hypertrophic cardiomyopathy: Role of age and clinical, electrocardiographic and hemodynamic features. Am. J. Cardiol..

[18] Geske J.B., Ommen S.R., Gersh B.J. (2018). Hypertrophic cardiomyopathy: Clinical update. JACC Heart Fail..

[19] Christiaans I., Van Engelen K., Van Langen I.M., Birnie E., Bonsel G.J., Elliott P., Wilde A.A. (2010). Risk stratification for sudden cardiac death in hypertrophic cardiomyopathy: Systematic review of clinical risk markers. Europace.

[20] Charron P., Dubourg O., Desnos M., Bennaceur M., Carrier L., Camproux A.-C., Isnard R., Hagege A., Langlard J.M., Bonne G. (1998). Clinical Features and Prognostic Implications of Familial Hypertrophic Cardiomyopathy Related to the Cardiac Myosin-Binding Protein C Gene. Circulation.

[21] Niimura H., Bachinski L.L., Sangwatanaroj S., Watkins H., Chudley A.E., McKenna W., Kristinsson A., Roberts R., Sole M., Maron B.J. (1998). Mutations in the Gene for Cardiac Myosin-Binding Protein C and Late-Onset Familial Hypertrophic Cardiomyopathy. N. Engl. J. Med..

[22] Schiavone W.A., Maloney J.D., Lever H.M., Castle L.W., Sterba R., Morant V. (1986). Electrophysiologic Studies of Patients with Hypertrophic Cardiomyopathy Presenting with Syncope of Undetermined Etiology. Pacing Clin. Electrophysiol..

[23] Barriales-Villa R., Centurión-Inda R., Fernández-Fernández X., Ortiz M.F., Pérez-Alvarez L., Rodríguez García I., Hermida-Prieto M., Monserrat L. (2010). Severe cardiac conduction disturbances and pacemaker implantation in patients with hypertrophic cardiomyopathy. Rev. Esp. Cardiol..

[24] Maron B.J., Shen W.-K., Link M.S., Epstein A.E., Almquist A.K., Daubert J.P., Bardy G.H., Favale S., Rea R.F., Boriani G. (2000). Efficacy of Implantable Cardioverter–Defibrillators for the Prevention of Sudden Death in Patients with Hypertrophic Cardiomyopathy. N. Engl. J. Med..

[25] Monserrat L., Elliott P.M., Gimeno J.R., Sharma S., Penas-Lado M., McKenna W.J. (2003). Non-sustained ventricular tachycardia in hypertrophic cardiomyopathy: An independent marker of sudden death risk in young patients. J. Am. Coll. Cardiol..

[26] Maron B.J., McKenna W.J., Danielson G.K., Kappenberger L.J., Kuhn H.J., Seidman C.E., Shah P.M., Spencer W.H., Spirito P., Cate F.J.T. (2003). American College of Cardiology/European Society of Cardiology Clinical Expert Consensus Document on Hypertrophic Cardiomyopathy A report of the American College of Cardiology Foundation Task Force on Clinical Expert Consensus Documents and the European Society of Cardiology Committee for Practice Guidelines. Eur. Heart J..

[27] Konno T., Hayashi K., Fujino N., Oka R., Nomura A., Nagata Y., Hodatsu A., Sakata K., Furusho H., Takamura M. (2015). Electrocardiographic QRS Fragmentation as a Marker for Myocardial Fibrosis in Hypertrophic Cardiomyopathy. J. Cardiovasc. Electrophysiol..

[28] Femenía F., Arce M., Van Grieken J., Trucco E., Mont L., Abello M., Merino J.L., Rivero-Ayerza M., Gorenek B., on behalf of Fragmented QRS in Hypertrophic Obstructive Cardiomyopathy (FHOCM) Study Investigators (2013). Fragmented QRS as a predictor of arrhythmic events in patients with hypertrophic obstructive cardiomyopathy. J. Interv. Card. Electrophysiol..

[29] Nienaber C.A., Gambhir S.S., Mody F.V., Ratib O., Huang S.C., Phelps M.E., Schelbert H.R. (1993). Regional myocardial blood flow and glucose utilization in symptomatic patients with hypertrophic cardiomyopathy. Circulation.

[30] Zyılmaz S., Püşüroğlu H. (2018). Assessment of the relationship between the ambulatory electrocardiography-based micro T-wave alternans and the predicted risk score of sudden cardiac death at 5 years in patients with hypertrophic cardiomyopathy. Anatol. J. Cardiol..

[31] Akboğa M.K., Gülcihan Balcı K., Yılmaz S., Aydın S., Yayla Ç., Ertem A.G., Ünal S., Balcı M.M., Balbay Y., Aras D. (2017). Tp-e interval and Tp-e/QTc ratio as novel surrogate markers for prediction of ventricular arrhythmic events in hypertrophic cardiomyopathy. Anatol. J. Cardiol..

[32] Bi X., Yang C., Song Y., Yuan J., Cui J., Hu F., Qiao S. (2020). Quantitative fragmented QRS has a good diagnostic value on myocardial fibrosis in hypertrophic obstructive cardiomyopathy based on clinical-pathological study. BMC Cardiovasc. Disord..

[33] Kang K.-W., Janardhan A.H., Jung K.T., Lee H.S., Lee M.-H., Hwang H.J. (2014). Fragmented QRS as a candidate marker for high-risk assessment in hypertrophic cardiomyopathy. Heart Rhythm.

[34] Savage D.D., Seides S.F., Maron B.J., Myers D.J., Epstein S.E. (1979). Prevalence of arrhythmias during 24-hour electrocardiographic monitoring and exercise testing in patients with obstructive and nonobstructive hypertrophic cardiomyopathy. Circulation.

[35] Elliott P.M., Gimeno Blanes J.R., Mahon N.G., Poloniecki J.D., McKenna W.J. (2001). Relation between severity of left-ventricular hypertrophy and prognosis in patients with hypertrophic cardiomyopathy. Lancet.

[36] Spirito P., Rapezzi C., Autore C., Bruzzi P., Bellone P., Ortolani P., Fragola P.V., Chiarella F., Zoni-Berisso M., Branzi A. (1994). Prognosis of asymptomatic patients with hypertrophic cardiomyopathy and nonsustained ventricular tachycardia. Circulation.

[37] Maron B.J., Savage D.D., Wolfson J.K., Epstein S.E. (1981). Prognostic significance of 24 hour ambulatory electrocardiographic monitoring in patients with hypertrophic cardiomyopathy: A prospective study. Am. J. Cardiol..

[38] McKenna W.J., England D., Doi Y.L., Deanfield J.E., Oakley C., Goodwin J.F. (1981). Arrhythmia in hypertrophic cardiomyopathy. I: Influence on prognosis. Br. Heart J..

[39] Greulich S., Seitz A., Herter D., Günther F., Probst S., Bekeredjian R., Gawaz M., Sechtem U., Mahrholdt H. (2021). Long-term risk of sudden cardiac death in hypertrophic cardiomyopathy: A cardiac magnetic resonance outcome study. Eur. Heart J. Cardiovasc. Imaging.

[40] Efthimiadis G.K., Parcharidou D.G., Giannakoulas G., Pagourelias E.D., Charalampidis P., Savvopoulos G., Ziakas A., Karvounis H., Styliadis I.H., Parcharidis G.E. (2009). Left Ventricular Outflow Tract Obstruction as a Risk Factor for Sudden Cardiac Death in Hypertrophic Cardiomyopathy. Am. J. Cardiol..

[41] Wang W., Lian Z., Rowin E.J., Maron B.J., Maron M.S., Link M.S. (2017). Prognostic Implications of Nonsustained Ventricular Tachycardia in High-Risk Patients with Hypertrophic Cardiomyopathy. Circ. Arrhythm. Electrophysiol..

[42] Sadoul N., Prasad K., Elliott P.M., Bannerjee S., Frenneaux M.P., McKenna W.J. (1997). Prospective prognostic assessment of blood pressure response during exercise in patients with hypertrophic cardiomyopathy. Circulation.

[43] Counihan P.J., Frenneaux M.P., Webb D.J., McKenna W.J. (1991). Abnormal vascular responses to supine exercise in hypertrophic cardiomyopathy. Circulation.

[44] Elliott P.M., Gimeno J.R., Tomé M.T., Shah J., Ward D., Thaman R., Mogensen J., McKenna W.J. (2006). Left ventricular outflow tract obstruction and sudden death risk in patients with hypertrophic cardiomyopathy. Eur. Heart J..

[45] Wang R.S., Rowin E.J., Maron B.J., Maron M.S., Maron B.A. (2023). A novel patient-patient network medicine approach to refine hypertrophic cardiomyopathy subgrouping: Implications for risk stratification. Cardiovasc. Res..

[46] Sinagra G., Carriere C., Clemenza F., Minà C., Bandera F., Zaffalon D., Gugliandolo P., Merlo M., Guazzi M., Agostoni P. (2020). Risk stratification in cardiomyopathy. Eur. J. Prev. Cardiol..

[47] Magrì D., Limongelli G., Re F., Agostoni P., Zachara E., Correale M., Mastromarino V., Santolamazza C., Casenghi M., Pacileo G. (2016). Cardiopulmonary exercise test and sudden cardiac death risk in hypertrophic cardiomyopathy. Heart.

[48] Masri A., Pierson L.M., Smedira N.G., Agarwal S., Lytle B.W., Naji P., Thamilarasan M., Lever H.M., Cho L.S., Desai M.Y. (2015). Predictors of long-term outcomes in patients with hypertrophic cardiomyopathy undergoing cardiopulmonary stress testing and echocardiography. Am. Heart J..

[49] Pelliccia A., Sharma S., Gati S., Bäck M., Börjesson M., Caselli S., Collet J.-P., Corrado D., Drezner J.A., Halle M. (2021). 2020 ESC Guidelines on sports cardiology and exercise in patients with cardiovascular disease. Eur. Heart J..

[50] Ho C.Y., Day S.M., Ashley E.A., Michels M., Pereira A.C., Jacoby D., Cirino A.L., Fox J.C., Lakdawala N.K., Ware J.S. (2018). Genotype and Lifetime Burden of Disease in Hypertrophic Cardiomyopathy: Insights from the Sarcomeric Human Cardiomyopathy Registry (SHaRe). Circulation.

[51] Kim H.Y., Park J.E., Lee S.-C., Jeon E.-S., On Y.K., Kim S.M., Choe Y.H., Ki C.-S., Kim J.-W., Kim K.H. (2020). Genotype-Related Clinical Characteristics and Myocardial Fibrosis and Their Association with Prognosis in Hypertrophic Cardiomyopathy. J. Clin. Med..

[52] Dimitrow P.P., Chojnowska L., Rudzinski T., Piotrowski W., Ziólkowska L., Wojtarowicz A., Wycisk A., Dabrowska-Kugacka A., Nowalany-Kozielska E., Sobkowicz B. (2010). Sudden death in hypertrophic cardiomyopathy: Old risk factors re-assessed in a new model of maximalized follow-up. Eur. Heart J..

[53] Velicki L., Jakovljevic D.G., Preveden A., Golubovic M., Bjelobrk M., Ilic A., Stojsic S., Barlocco F., Tafelmeier M., Okwose N. (2020). Genetic determinants of clinical phenotype in hypertrophic cardiomyopathy. BMC Cardiovasc. Disord..

[54] Elliott P.M., Anastasakis A., Borger M.A., Borggrefe M., Cecchi F., Charron P., Hagege A.A., Lafont A., Limongelli G., Mahrholdt H. (2014). 2014 ESC Guidelines on diagnosis and management of hypertrophic cardiomyopathy: The Task Force for the Diagnosis and Management of Hypertrophic Cardiomyopathy of the European Society of Cardiology (ESC). Eur. Heart J..

[55] Turvey L., Augustine D.X., Robinson S., Oxborough D., Stout M., Smith N., Harkness A., Williams L., Steeds R.P., Bradlow W. (2021). Transthoracic echocardiography of hypertrophic cardiomyopathy in adults: A practical guideline from the British Society of Echocardiography. Echo Res. Pract..

[56] Habib M., Hoss S., Rakowski H. (2019). Evaluation of Hypertrophic Cardiomyopathy: Newer Echo and MRI Approaches. Curr. Cardiol. Rep..

[57] Weissler-Snir A., Dorian P., Rakowski H., Care M., Spears D. (2021). Primary prevention implantable cardioverter-defibrillators in hypertrophic cardiomyopathy-Are there predictors of appropriate therapy?. Heart Rhythm.

[58] Watson R.M., Schwartz J.L., Maron B.J., Tucker E., Rosing D.R., Josephson M.E. (1987). Inducible polymorphic ventricular tachycardia and ventricular fibrillation in a subgroup of patients with hypertrophic cardiomyopathy at high risk for sudden death. J. Am. Coll. Cardiol..

[59] Kuck K.-H., Kunze K.-P., Schluter M., Nienaber C.A., Costard A. (1988). Programmed electrical stimulation in hypertrophic cardiomyopathy. Results in patients with and without cardiac arrest or syncope. Eur. Heart J..

[60] Geibel A., Brugada P., Zehender M., Stevenson W., Waldecker B., Wellens H.J. (1987). Value of programmed electrical stimulation using a standardized ventricular stimulation protocol in hypertrophic cardiomyopathy. Am. J. Cardiol..

[61] Gatzoulis K.A., Georgopoulos S., Antoniou C.-K., Anastasakis A., Dilaveris P., Arsenos P., Sideris S., Tsiachris D., Archontakis S., Sotiropoulos E. (2018). Programmed ventricular stimulation predicts arrhythmic events and survival in hypertrophic cardiomyopathy. Int. J. Cardiol..

[62] Efthimiadis G.K., Pliakos C., Pagourelias E.D., Parcharidou D.G., Giannakoulas G., Kamperidis V., Hadjimiltiades S., Karvounis C., Gavrielidis S., Styliadis I.H. (2009). Identification of high risk patients with hypertrophic cardiomyopathy in a northern Greek population. Cardiovasc. Ultrasound.

[63] Ommen S.R., Mital S., Burke M.A., Day S.M., Deswal A., Elliott P., Evanovich L.L., Hung J., Joglar J.A., Kantor P. (2020). 2020 AHA/ACC Guideline for the Diagnosis and Treatment of Patients with Hypertrophic Cardiomyopathy: A Report of the American College of Cardiology/American Heart Association Joint Committee on Clinical Practice Guidelines. Circulation.

[64] Maron B.J., Casey S.A., Chan R.H., Garberich R.F., Rowin E.J., Maron M.S. (2015). Independent Assessment of the European Society of Cardiology Sudden Death Risk Model for Hypertrophic Cardiomyopathy. Am. J. Cardiol..

[65] Vriesendorp P.A., Schinkel A.F., Liebregts M., Theuns D.A., van Cleemput J., Ten Cate F.J., Willems R., Michels M. (2015). Validation of the 2014 European Society of Cardiology guidelines risk prediction model for the primary prevention of sudden cardiac death in hypertrophic cardiomyopathy. Circ. Arrhythm. Electrophysiol..

[66] Norrish G., Ding T., Field E., Ziólkowska L., Olivotto I., Limongelli G., Anastasakis A., Weintraub R., Biagini E., Ragni L. (2019). Development of a novel risk prediction model for sudden cardiac death in childhood hypertrophic cardiomyopathy (HCM risk-kids). JAMA Cardiol..

[67] Norrish G., Qu C., Field E., Cervi E., Khraiche D., Klaassen S., Ojala T.H., Sinagra G., Yamazawa H., Marrone C. (2022). External validation of the HCM Risk-Kids model for predicting sudden cardiac death in childhood hypertrophic cardiomyopathy. Eur. J. Prev. Cardiol..

[68] Sorajja P. (2017). Alcohol Septal Ablation for Obstructive Hypertrophic Cardiomyopathy: Alcohol Septal Ablation for Obstructive Hypertrophic Cardiomyopath. J. Am. Coll. Cardiol..

[69] Olivotto I., Oreziak A., Barriales-Villa R., Abraham T.P., Masri A., Garcia-Pavia P., Saberi S., Lakdawala N.K., Wheeler M.T., Owens A. (2020). Mavacamten for treatment of symptomatic obstructive hypertrophic cardiomyopathy (EXPLORER-HCM): A randomised, double-blind, placebo-controlled, phase 3 trial. Lancet.

[70] Sánchez-Somonte P., Quinto L., Garre P., Zaraket F., Alarcón F., Borràs R., Caixal G., Vázquez S., Prat S., Ortiz-Perez J.T. (2021). Scar channels in cardiac magnetic resonance to predict appropriate therapies in primary prevention. Heart Rhythm.

[71] Mancio J., Pashakhanloo F., El-Rewaidy H., Jang J., Joshi G., Csecs I., Ngo L., Rowin E., Manning W., Maron M. (2022). Machine learning phenotyping of scarred myocardium from cine in hypertrophic cardiomyopathy. Eur. Heart J. Cardiovasc. Imaging.

